# Recognition of a Clickable Abasic Site Analog by DNA Polymerases and DNA Repair Enzymes

**DOI:** 10.3390/ijms232113353

**Published:** 2022-11-01

**Authors:** Anton V. Endutkin, Anna V. Yudkina, Timofey D. Zharkov, Daria V. Kim, Dmitry O. Zharkov

**Affiliations:** 1 SB RAS Institute of Chemical Biology and Fundamental Medicine, 8 Lavrentieva Ave., Novosibirsk 630090, Russia; 2 Department of Natural Sciences, Novosibirsk State University, 2 Pirogova Street, Novosibirsk 630090, Russia

**Keywords:** click chemistry, AP site, DNA repair, AP endonucleases, DNA glycosylases, translesion synthesis, DNA polymerases

## Abstract

Azide–alkyne cycloaddition (“click chemistry”) has found wide use in the analysis of molecular interactions in living cells. 5-ethynyl-2-(hydroxymethyl)tetrahydrofuran-3-ol (EAP) is a recently developed apurinic/apyrimidinic (AP) site analog functionalized with an ethynyl moiety, which can be introduced into cells in DNA constructs to perform labeling or cross-linking in situ. However, as a non-natural nucleoside, EAP could be subject to removal by DNA repair and misreading by DNA polymerases. Here, we investigate the interaction of this clickable AP site analog with DNA polymerases and base excision repair enzymes. Similarly to the natural AP site, EAP was non-instructive and followed the “A-rule”, directing residual but easily detectable incorporation of dAMP by *E. coli* DNA polymerase I Klenow fragment, bacteriophage RB69 DNA polymerase and human DNA polymerase β. On the contrary, EAP was blocking for DNA polymerases κ and λ. EAP was an excellent substrate for the major human AP endonuclease APEX1 and *E. coli* AP exonucleases Xth and Nfo but was resistant to the AP lyase activity of DNA glycosylases. Overall, our data indicate that EAP, once within a cell, would represent a replication block and would be removed through an AP endonuclease-initiated long-patch base excision repair pathway.

## 1. Introduction

The concept of bioorthogonality, which appeared in the field of synthetic biology, refers to any chemical reaction that can occur within living systems without interfering with natural biochemical processes [1,2]. For example, regulatory networks based on genetic elements foreign to a given cell and proteins binding to them can be bioorthogonal [3,4]. On the other hand, chemical groups in biomolecules that can enter into highly specific reactions that do not affect other components of the cell, or such reactions themselves, are considered bioorthogonal [5,6]. The copper(I)-catalyzed cycloaddition reaction between azides and alkynes with the formation of 1,2,3-triazoles [7,8] turned out to be so regioselective and efficient even under the mildest conditions that it gave rise to an entire field of “click chemistry”, culminating in the 2022 Nobel Prize in Chemistry for its discovery. The azide–alkyne cycloaddition and [4 + 2] cycloaddition (Diels–Alder reaction) are good examples of biorthogonal reactions widely used in synthetic biology [9,10,11]. The range of bioorthogonal reactions and methods of their implementation in living cells is growing steadily.

**Figure 1:**
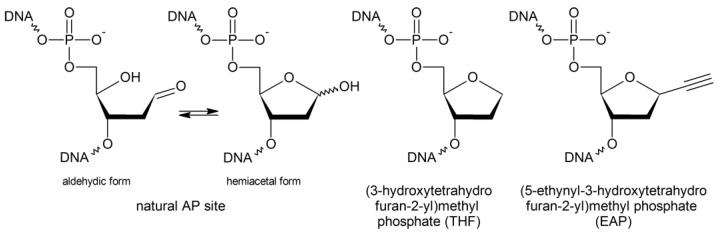
Structures of the natural AP site and its synthetic analogs.

Impressive advances in the synthetic nucleic acid chemistry has put these macromolecules among the most convenient ones for carrying out bioorthogonal reactions in situ. While the introduction of non-natural reactive groups at exact positions into protein molecules is possible but requires extensive molecular cloning manipulation, DNA and RNA with many desired functional modifications can be easily obtained chemically. From the very first days of using synthetic nucleic acids to work in living cells, they were modified to increase the resistance to nucleases, which, in fact, is conferring bioorthogonality, i.e., reducing interactions with the cellular machinery. In addition to chemically active and inhibitory groups, synthetic nucleic acids used in cells are often equipped with substituents that modulate their complementary properties, thermodynamic stabilization, add reporter groups, etc. The use of click chemistry to modify DNA has been somewhat delayed by DNA-damaging properties of copper ions, usually leading to strand breaks [12]. However, these difficulties have now been overcome by the use of ligands that stabilize copper(I), and click reactions are successfully used for highly efficient postsynthetic functionalization of alkyne-modified DNA nucleobases or non-nucleotide linkers [13,14]. In particular, (5-ethynyl-3-hydroxytetrahydrofuran-2-yl)methyl phosphate (1-ethynyl-2-deoxy-β-D- ribofuranose; abasic click reactive nucleoside; EAP; Figure 1) has found its use in studies of in situ crosslinking and adding functional groups in place of nucleobases [15,16,17].

Chemical modification of DNA in living cells is tightly controlled by DNA repair and DNA damage response systems [18]. Any non-canonical nucleotide in DNA, with the exception of a very small number of natural modifications (e.g., 5-methylcytosine or 5-hydroxymethylcytosine), is perceived as a lesion and removed. Small non-bulky lesions are usually removed through the base excision repair (BER) pathway [18,19,20]. If a nucleobase is damaged, BER is initiated by one of many DNA glycosylases (eleven are known in human cells, eight, in *E. coli*), which hydrolyze the *N*-glycosidic bond of the target nucleotide. The baseless deoxyribose formed is commonly known as an apurinic/apyrimidinic (AP) site, or an abasic site (Figure 1). Alternatively, the AP site itself may be a primary lesion after spontaneous base loss, which may be further complicated with chemical conversion (e.g., oxidation) of the AP site. Whether glycosylase-produced or spontaneous, AP sites are processed by AP endonucleases that hydrolyze the phosphodiester bond 5′ to the AP site. Some glycosylases can also nick DNA at AP sites but do it by β-elimination 3′ to the AP site rather than by hydrolysis; this reaction is usually referred to as an AP lyase activity. Incorporation of a normal dNMP by a DNA polymerase followed by ligation complete the round of BER. If not repaired, AP sites are often more harmful than damaged bases due to their high mutagenicity as non-instructive lesions and the tendency to produce DNA breaks and trap nucleophilic molecules [18,21]. AP endonucleases also recognize many types of synthetic abasic units, such as (3-hydroxytetrahydrofuran-2-yl)methyl phosphate (THF; Figure 1), which is often used as an experimental substitute for the natural AP site due to better stability.

EAP, a synthetically available clickable abasic DNA unit, is likely to be recognized by the BER system. Its overall size and the lack of base-pairing properties resemble the AP site or naturally occurring AP site derivatives processed by AP endonucleases. On the other hand, electron-withdrawing properties of the alkyne moiety could make EAP sensitive to DNA glycosylases due to an increased positive charge at C1′, as was observed for 1-*O*-methyl-2-deoxyribose [22]. As for their coding properties, EAP sites may resemble natural or synthetic abasic sites, which often direct low-efficiency dAMP incorporation. Thus, it was of interest to investigate the interaction of this clickable abasic site analog with DNA polymerases and BER enzymes.

## 2. Results

### 2.1. DNA Polymerases Preferentially Incorporate dAMP Opposite to EAP

Abasic DNA units of various chemical nature are by definition non-instructive but tend to direct incorporation of dAMP; this preference is known as the “A-rule” [23,24]. To address the behavior of DNA polymerases encountering EAP in DNA, we have used four enzymes representative of four structural families: Klenow fragment of *E. coli* DNA polymerase I (KF; Family A; 3′→5′ exonuclease-deficient mutant was used), bacteriophage RB69 DNA polymerase (RBpol; Family B; 3′→5′ exonuclease-deficient mutant was used), human DNA polymerases β (POLβ) and λ (POLλ; Family X) and human DNA polymerase κ (POLκ; Family Y).

**Figure 2:**
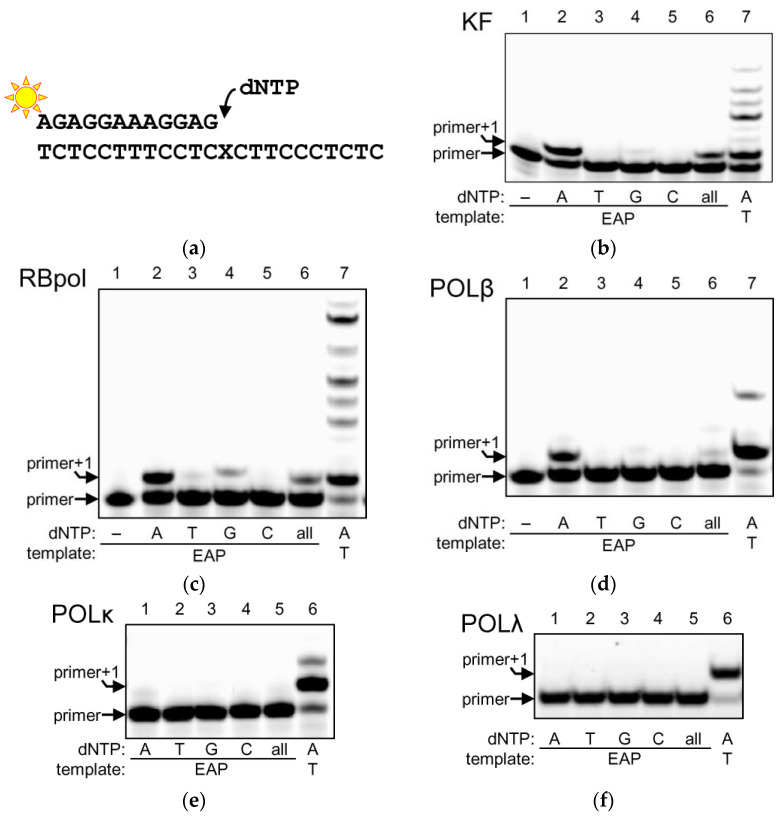
Incorporation of dNMPs by DNA polymerases opposite EAP. (**a**) Fluorescently labeled primer-template substrate used in the experiments. (**b**–**f**) Incorporation of dNMPs by KF (**b**), RBpol (**c**), POLβ (**d**), POLκ (**e**) and POLλ (**f**). The nature of the substrates is indicated below the representative gel images.

DNA polymerase I has a specialized role in completing the bacterial lagging strand synthesis and is the major BER DNA polymerase in *E. coli* [25]. When presented with the EAP substrate (Figure 2a) and individual dNTPs, KF strictly followed the A-rule efficiently incorporating dAMP opposite to the abasic unit (Figure 2b). Other than that, only a trace dGMP incorporation was observed. If a mixture of all dNTPs was used, we did not see extension beyond the EAP site, indicating that KF always terminates the synthesis after dAMP incorporation.

RBpol is a highly processive, high-fidelity replicative phage DNA polymerase. Since it belongs to the same Family B as human DNA polymerases α, δ and ε, RBpol is often used as an accessible model of polymerase biochemistry relevant to human replication machinery [26]. RBpol incorporated dAMP and, less efficiently, dGMP, with a trace amount of dTMP (Figure 2c). As with KF, no extension beyond the modified site was observed with the mixture of all dNTPs; thus, EAP is also a blocking lesion for RBol. Note that RBPol and KF are processive enzymes and tend to stay on DNA after incorporating a nucleotide, so in the presence of high dNTP concentrations and with the proofreading exonuclease function disabled, they can misincorporate dNMPs opposite non-complementary bases [27], as can be seen in Figure 2b,c, lane 7. POLβ and POLκ also have some ability to support this reaction. No such spurious extension was observed when EAP was in the template, consistent with strong blocking properties of this modification.

POLβ and POLλ belong to Family X, which includes eukaryotic DNA polymerases specialized in DNA repair and recombination [28]. Whereas POLβ is the major BER polymerase in higher eukaryotes, POLλ seems to play only a back-up role in BER and is mostly involved with DNA ends processing in the non-homologous end joining pathway of double-strand breaks repair. When presented with an EAP-containing template, POLβ incorporated almost exclusively dAMP (Figure 2d). Interestingly, POLβ was the only polymerase that extended the template after the first insertion in the presence of all dNMPs, albeit with a low efficiency (Figure 2d). POLλ, on the other hand, showed no insertion whatsoever (Figure 2f).

Finally, POLκ is a translesion DNA polymerase specialized in the bypass of damaged nucleotides in the DNA damage tolerance pathway [29]. Notably, POLκ incorporates dNMPs opposite an abasic site with considerable efficiency [30,31,32,33]. However, with EAP in the template, we observed only very low incorporation of dAMP and no extension (Figure 2e).

**Figure 3:**
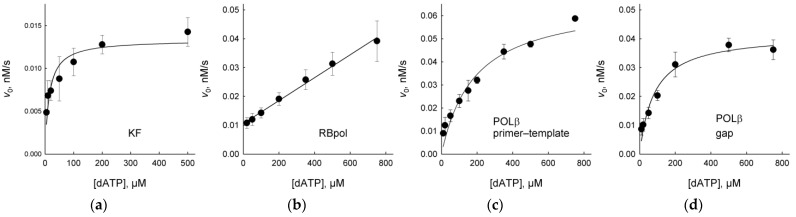
Kinetic curves of DNA polymerases incorporating dAMP opposite the template EAP. (**a**), KF; (**b**), RBpol; (**c**), POLβ with a primer-template substrate; (**d**), POLβ with a gapped substrate. Mean ± S.D. of three independent experiments is shown.

As can be seen from Figure 2b–d, when KF, RBpol and POLβ were presented with a 500 μM mixture of dNTPs, in which dATP constituted only 25%, the incorporation was lower than with 500 μM dATP. This may reflect competition between dNTPs or an incomplete saturation of the enzyme by dATP when the active site is occupied with a non-instructive nucleotide. To characterize DNA polymerase interactions with EAP in a more quantitative fashion, we have determined steady-state kinetic parameters of incorporation of the preferred dAMP nucleotide opposite EAP in the template by KF, RBpol and POLβ (Figure 3, Table 1). Due to the very low activity of POLκ and POLλ, these polymerases could not be evaluated. Table 1 lists the Michaelis constant *K*_M_, the catalytic constant *k*_cat_ and the specificity constant *k*_cat_/*K*_M_ together with the same parameters reported for the natural AP site and for T in exactly the same oligonucleotide sequence context as used here [34]. Predictably, EAP and AP were much worse templates than T in all cases, yet there were some notable differences in the recognition of template EAP by KF, RBpol and POLβ. Both KF and POLβ were significantly more proficient in the insertion of dAMP opposite EAP compared with the regular AP site in the primer-template substrate. KF had similar *K*_M_ for dAMP opposite both template EAP and AP sites, but the *k*_cat_ value was about an order of magnitude higher for the incorporation opposite EAP. POLβ uses the AP site in the primer-template system very inefficiently, although it can bypass abasic lesions in a gapped context when a downstream strand is also present [34,35,36]. Nevertheless, *K*_M_ and *k*_cat_ could be determined for dAMP incorporation opposite EAP, being 43-fold higher and 14-fold lower, respectively, compared to undamaged DNA. Since a 1-nt gap is a preferred type of substrate for POLβ [37,38], we have also determined its kinetics on this substrate. For the gapped EAP duplex, the efficiency (in terms of *k*_cat_/*K*_M_) was slightly better than for the primer-template but was 2.9-fold lower than for the dAMP incorporation opposite the natural AP site. Interestingly, a comparison of *K*_M_ and *k*_cat_ for the gapped AP and EAP substrates reveals that, with EAP, dATP binding was 28-fold worse, while the reaction rate was an order of magnitude better. In contrast to KF and POLβ, RBpol incorporated dAMP opposite EAP 63-fold less efficiently than opposite the natural AP site and >19,000-fold less efficiently than opposite T. Saturation or RBpol with the dATP could not be achieved.

### 2.2. EAP Is Processed by AP Endonucleases

AP endonucleases belong to two different structural superfamilies. The major human AP endonuclease, APEX1, and its *E. coli* homolog exonuclease III (Xth) are members of the large exonuclease–endonuclease–phosphatase superfamily [40,41]. *E. coli* also has another AP endonuclease, endonuclease IV (Nfo), which belongs to the TIM barrel superfamily [40,41]. Despite their completely different structures, APEX1/Xth and Nfo perform identical functions: they hydrolyze DNA 5′ to the natural (aldehydic) AP site as well as many naturally occurring AP site modifications and synthetic abasic linkers [40,42]. Some modifications, however, render DNA resistant to the cleavage; for example, AP endonucleases cannot process *O*-alkoxyimino derivatives of AP sites [43,44].

**Figure 4:**
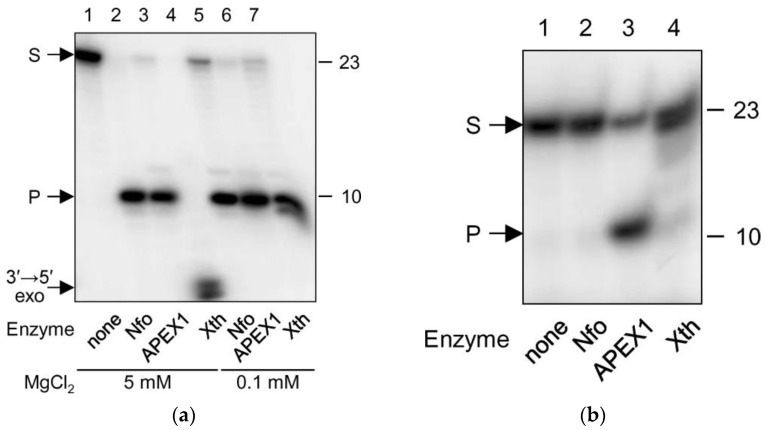
Cleavage of EAP by AP endonucleases Nfo, APEX1 and Xth. (**a**) Cleavage of EAP in double-stranded DNA (EAP:G). Reaction mixtures contained 5 mM (lanes 1–4) or 0.1 mM (lanes 5–7) MgCl_2_. (**b**) Cleavage of EAP in single-stranded DNA (5 mM MgCl_2_). The nature of the enzyme is indicated below the gel image. Arrows indicate: S, 23-mer substrate; P, 10-mer product, 3′→5′ exo, product of exonucleolytic degradation.

In order to assess the substrate properties of EAP for AP endonucleases, we have treated duplex oligonucleotides containing an EAP:G pair with APEX1, Xth or Nfo (Figure 4a). All three enzymes efficiently cleaved this substrate forming a product that migrated with a higher mobility during gel electrophoresis. Xth additionally degraded this product due to its robust 3′→5′-exonuclease activity (Figure 4a, lane 4); however, this reaction was nearly eliminated if Mg^2+^ was reduced to submillimolar levels (Figure 4b, lanes 4 and 7). As APEX1 was reported to possess AP endonuclease activity on single-stranded DNA [45], albeit 20-fold lower one than on double-stranded substrates, we have also checked the activity of all three enzymes on single-stranded EAP-containing oligonucleotides. Consistent with the literature, APEX1 hydrolyzed such substrates but with significantly lower efficiency (Figure 4a), whereas Xth and Nfo lacked such activity (not shown).

**Figure 5:**
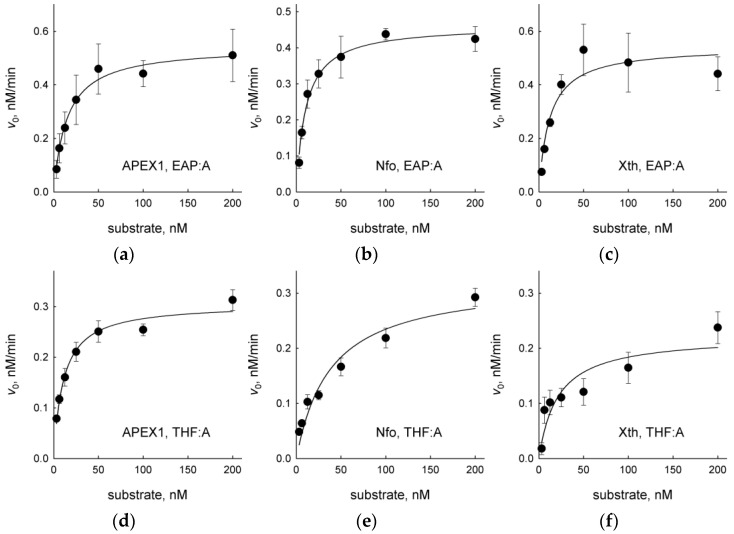
Kinetic curves of AP endonucleases cleaving EAP:A (**a**–**c**) or THF:A substrates (**d**–**f**). (**a**,**d**), APEX1; (**b**,**e**), Nfo; (**c**,**f**), Xth. Mean ± S.D. of three independent experiments is shown.

We have also compared steady-state kinetic parameters of APEX1, Nfo and Xth on duplex substrates containing EAP or THF, a widely used AP site analog resistant to spontaneous β-elimination [46,47,48,49,50,51] (Figure 5, Table 2). As can be seen from Table 2, EAP was an even better substrate for Nfo and Xth in terms of the specificity constant, *k*_cat_/*K*_M_. This was due to both lower *K*_M_ and higher *k*_cat_ for the EAP-containing DNA. APEX1 recognized EAP and THF in DNA with approximately the same efficiency.

### 2.3. EAP Is Resistant to DNA Glycosylases

DNA glycosylases recognize and remove damaged DNA bases or, in some cases, normal bases placed in a wrong base-pairing context [18,52]. Genomes of all living organisms encode a set of DNA glycosylases specific to certain types of base lesions; for example, *E. coli* possesses nine DNA glycosylases, and human cells eleven. These enzymes belong to several structural superfamilies and, based on their mechanism, can be divided into monofunctional and bifunctional. Monofunctional DNA glycosylases use a water molecule for the nucleophilic attack at C1′ of the damaged nucleotide to displace the target nucleobase and produce an AP site. Bifunctional DNA glycosylases use an enzyme’s amino group as a nucleophile, form a Schiff base-type covalent reaction intermediate and catalyze β-elimination of the 3′-phosphate (AP lyase reaction) yielding a single-strand break or a one-base gap in DNA [53,54]. The latter group also quite efficiently cleaves natural AP sites using the same reaction chemistry but cannot process THF or other non-aldehydic abasic units [55,56,57,58,59].

**Figure 6:**
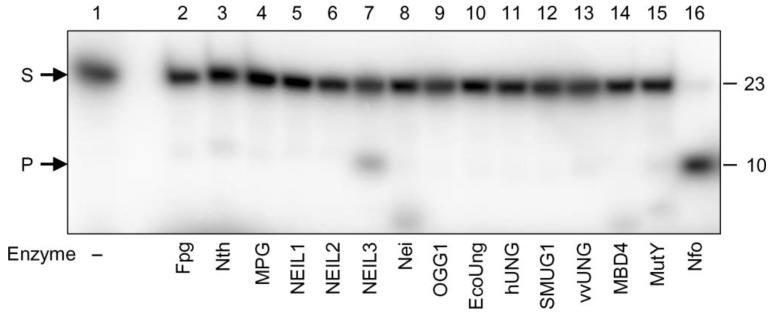
Resistance of EAP to DNA glycosylases. A representative gel illustrating the reaction of EAP with double-stranded DNA (EAP:C) is shown. The nature of the enzymes is indicated below the gel image. Arrows indicate: S, 23-mer substrate; P, 10-mer product.

We have screened a collection of DNA glycosylases available in our laboratory for their ability to cleave EAP-containing oligonucleotide substrates. The panel, encompassing nearly the full known substrate specificity range of DNA glycosylases, included *E. coli* Fpg, MutY (catalytic p25 domain), Nei, Nth and Ung, human MBD4 (catalytic domain), MPG, NEIL1, NEIL2, NEIL3, OGG1, SMUG1 and UNG and vaccinia virus D4 proteins. Of these, Fpg, Nei, Nth, NEIL1, NEIL2, NEIL3 and OGG1 are bifunctional and cleave natural AP sites by means of their AP lyase activity, and the rest are monofunctional. As substrates, we used duplexes in which EAP was placed opposite to any of the four canonical bases, as well as single-stranded oligonucleotides. Although in a few cases at a large enzyme excess (20-fold) and long incubation times (1 h) we observed some low-level spurious degradation, no DNA glycosylase was able to cleave any of the EAP substrates with an appreciable efficiency (Figure 6). Heating with piperidine, which breaks DNA at natural AP sites, had no effect either alone or after the glycosylase treatment. We conclude that EAP is resistant to the action of DNA glycosylases irrespective of their mechanism.

## 3. Discussion

EAP is a recent addition to the list of synthetic AP site analogs, which includes THF [46] and reduced AP site [60], often used as model abasic lesions due to their resistance to β-elimination, and less common cyclopentane [61], carbocyclic AP sites [62], pyrrolidines [63,64], acyclic diols and oligoethylene glycols with varying spacer lengths [65,66,67], etc. EAP, designed as a tool for conjugation of various moieties to C1′ of the DNA backbone through click chemistry [15,16], can be introduced into living cells within artificial DNA constructs to perform labeling or cross-linking in situ [68,69]. However, as EAP is a non-natural nucleoside, it could be subject to removal by DNA repair and to bypass by DNA polymerases in the cell.

With respect to its DNA polymerase-templating properties, EAP turned out to resemble the natural AP site and its synthetic analogs but had some distinctive features regarding polymerases of different families. EAP was a much better substrate for the Family A KF and Family X POLβ than the natural AP site. At the same time, Family B RBpol bypassed EAP ~2 orders of magnitude less efficiently than the natural AP site. Since Family B also includes the major human replicative DNA polymerases α, δ and ε, one may expect that in the cell EAP would represent a strong replication block and would need to be repaired lest it triggers fork collapse. Perhaps most unexpectedly, EAP could not be bypassed by Family Y POLκ, a specialized translesion polymerase with a relaxed active site that allows it to insert dNMPs opposite damaged nucleotides including natural and synthetic abasic sites [30,31,32,33].

When DNA polymerases were able to incorporate a dNMP opposite EAP, it was predominantly dAMP. Therefore, similarly to the natural AP site and THF, EAP follows the A-rule. Interestingly, in different DNA polymerases, the A-rule is instigated through different mechanisms. KF is suggested to present a highly conserved Tyr residue in place of the template nucleotide if the base is missing, which forms a good steric pair with the incoming dATP [70,71]. RBpol, on the other hand, mostly relies on the preferential stacking interactions between the dATP and the planar base pair system at the primer-template junction [72,73], while in POLβ, the preference for dATP selection is due to the kinetics of polymerase closing [74]. Obviously, the interactions of EAP with the polymerases’ active sites are similar enough to the interactions of AP or THF that these structural mechanisms of incoming dNTP selection could also operate for EAP.

The repair of EAP appears to follow the same pathway as the AP endonuclease-dependent repair of AP site analogs resistant to β-elimination. In mammalian cells, THF is repaired strictly through the long-patch BER subpathway initiated by APEX1 and requiring displacement DNA synthesis and flap removal by FEN1 [75]. A similar flap processing pathway operates in bacteria, although its relevance to BER is less well established [76]. EAP was comparable to or even better than THF in terms of its recognition and processing by APEX1 and two *E. coli* AP endonucleases, Xth and Nfo. Presumably, once inside the living cell, EAP would be quickly removed from DNA by BER. There is also accumulating evidence that nucleotide excision repair can process abasic lesions, including those resistant to BER [77,78]. Thus, despite the ability of EAP to serve as a platform for conjugation immediately to the DNA backbone, the time window of its use in situ is likely limited compared to base-containing clickable nucleotides.

## 4. Materials and Methods

### 4.1. Enzymes

Human APEX1 [79], NEIL1 [80], NEIL2 [81], MBD4 [82], OGG1 [83], UNG [84], POLκ (catalytic core, residues 1–560) [85], POLλ (catalytic core, residues 242–575) [86], *E. coli* Fpg [87], KF exo^−^ [88], MutY [89], Nei [90], Nfo [91], Nth [92], phage RB69 DNA polymerase exo^−^ [93] and vaccinia virus UNG [84] were purified essentially as described (Supplementary Figures S1 and S2). Human MPG, NEIL3 and SMUG1 proteins were a kind gift from Dr. Murat Saparbaev (Institut Gustave Roussy, France). *E. coli* Xth and Ung were purchased from SibEnzyme (Novosibirsk, Russia). Human POLβ cloning and purification is described below.

### 4.2. POLβ Cloning and Purification

Coding sequence of POLβ codon-optimized for expression in *E. coli* was synthesized *de novo* by Gene Universal (Newark, DE, USA) and confirmed by Sanger sequencing. The sequence was subcloned into the bacterial expression vector pET-24b (Merck Millipore, Burlington, MA, USA) at NdeI/XhoI sites. The plasmid was subsequently introduced into the *E. coli* Rossetta 2(DE3) strain (Merck Millipore). One liter of LB medium was inoculated with 5 mL overnight culture containing the expression plasmid and 100 μg/mL of kanamycin. The cells were grown with vigorous shaking at 37 °C to A_600_ = 0.8, isopropyl-β-D-thiogalactopyranoside was added to 1 mM, and the growth continued for 4 h at 37 °C. The cells were harvested by centrifugation at 12,000× *g* at 4 °C for 20 min and stored at −72 °C. Before the purification, the pellet was thawed on ice in 40 mL of buffer A consisting of 20 mM Na phosphate (pH 7.5), 5% glycerol, 1 mM ethylenediaminetetraacetic acid (EDTA), 1 mM dithiothreitol (DTT) and supplemented with 500 mM NaCl and 1 mM phenylmethylsulfonyl fluoride. The cells were sonicated, and the lysate was cleared by centrifugation at 12,000× *g* at 4 °C for 30 min. The supernatant was loaded onto a 5-mL Q Sepharose HiTrap column (GE Healthcare, Chicago, IL, USA) previously equilibrated in Buffer A supplemented with 500 mM NaCl. The flowthrough containing POLβ was diluted with four volumes of Buffer A and loaded onto a 5-mL heparin Sepharose HiTrap column (GE Healthcare) previously equilibrated in Buffer A supplemented with 100 mM NaCl. After several washing steps with Buffer A containing increasing concentrations of NaCl, POLβ eluted at 400 mM NaCl. The fractions containing POLβ were pooled, diluted with five volumes of Buffer A, and loaded onto a 1-mL MonoS column (GE Healthcare) previously equilibrated in Buffer A supplemented with 100 mM NaCl. The column was washed with the same buffer, and POLβ was eluted by an NaCl gradient at ~400 mM NaCl. Fractions contains >90% homogeneous protein were pooled and dialyzed against the buffer containing 20 mM Na phosphate (pH 7.5), 50% glycerol, 1 mM EDTA, 1 mM DTT, and 100 mM NaCl and stored at −20 °C. The protein tested under the standing-start assay conditions (see below) in the absence of dNTP did not demonstrate any noticeable exonuclease activity.

### 4.3. Oligonucleotides

Oligonucleotides (Table 3) were synthesized in-house from commercially available phosphoramidites (Glen Research, Sterling, VA, USA) on an ASM800 automatic synthesizer (Biosset, Russia) according to the standard 2-cyanoethyl phosphoramidite protocol and purified by reverse-phase HPLC on a PRP-1 C18 column (Hamilton, Reno, NV, USA). The EAP-containing oligonucleotide was synthesized in the same way and cleaved from the solid support with the 1:1 ammonia/methylamine solution at 65 °C for 10 min. For the polymerase reactions, the fluorescein-labeled primer was annealed to a 2-fold molar excess of the complementary strand and, if necessary, the downstream strand. For the cleavage reactions, the EAP oligonucleotide was labeled using γ[^32^P]ATP (SB RAS ICBFM Laboratory of Biotechnology, Novosibirsk, Russia) and phage T4 polynucleotide kinase (Biosan, Novosibirsk, Russia) according to the manufacturer’s protocol, desalted on an Isolute C18 sorbent (Biotage, Uppsala, Sweden) and annealed to a 2-fold excess of the complementary strand.

### 4.4. Standing-Start DNA Polymerase Assay

The reaction mixture (10 µL) contained 50 mM Tris-HCl (pH 7.5), 5 mM MgCl_2_ (10 mM for POLβ), 1 mM DTT, 100 nM primer-template, 500 µM dNTP (A, T, G, C, or an equimolar mixture of all) and one of the DNA polymerases (2 nM KF, 10 nM RBpol, 10 nM POLβ, 5 nM POLκ, or 10 nM POLλ). The reaction was allowed to proceed for 10 min at 25 °C and stopped by adding an equal volume of the stop solution (20 mM EDTA in formamide) and heating at 95 °C for 2 min. The reaction products were resolved by electrophoresis in 20% polyacrylamide gel/7.2 M urea and visualized using a Typhoon FLA 9500 phosphorimager (GE Healthcare) in the fluorescence detection mode.

### 4.5. DNA Polymerase Steady-State Kinetics

The reaction mixtures were as described above except dATP in increasing concentrations (5–500 μM for KF, 20–750 µM for RBpol, 10–750 µM for POLβ) was the only dNTP. The concentrations of DNA polymerase for each substrate was optimized to give less than 30% insertion of the first nucleotide in 10 min at 25 °C: 0.5 nM KF or 10 nM RBpol and POLβ. The reactions were carried out and analyzed as described above. The imaged gels were quantified using Quantity One v4.6.3 (Bio-Rad Laboratories, Hercules, CA, USA). The reaction velocity vs. substrate concentration data were fitted by nonlinear regression to the Michaelis–Menten equation using SigmaPlot v11.0 (Systat Software, Chicago, IL, USA). All reported constants are derived from three independent experiments.

### 4.6. AP Endonuclease and DNA Glycosylase Assays

The reaction mixture (10 µL) contained 50 mM Tris-HCl (pH 7.5), 100 mM NaCl, either 5 mM or 0.1 mM MgCl_2_ (for AP endonucleases) or 1 mM EDTA (for DNA glycosylases), 1 mM DTT, 50 nM ^32^P-labeled oligonucleotide substrate (EAP opposite A, T, G, C or single-stranded) and 1 μM enzyme. The reaction was allowed to proceed for 1 h at 37 °C. The reactions with AP endonucleases were terminated by adding 5 μL of the stop solution (see above) containing 0.1% bromophenol blue and 0.1% xylene cyanol and heating for 3 min at 95 °C. The reactions with DNA glycosylases were terminated by adding 1 μL of 1 M NaOH and heating for 1 min at 95 °C, after which they were neutralized with an equimolar amount of HCl and mixed with 5 μL of the dye-containing stop solution. The reaction products were separated and visualized as described above, but in the ^32^P detection mode.

### 4.7. AP Endonuclease Steady-State Kinetics

The reaction mixtures were as described above for AP endonucleases except the DNA duplex was taken in increasing concentrations (3–200 nM), and the reaction time was 10 min. The concentrations of AP endonucleases were optimized to give less than 15% cleavage in 10 min at 37 °C: 12.5 pM APEX1, 175 pM Nfo, and 3 pM Xth. The reactions were carried out and analyzed as described above. The reaction velocity vs. substrate concentration data were fitted by nonlinear regression to the Michaelis-Menten equation. All reported constants are derived from 3–4 independent experiments.

## Figures and Tables

**Table 1:** Kinetic parameters of the insertion of dAMP opposite abasic and undamaged template nucleotides.

Enzyme	Template Nucleotide	*K*_M_, μM	*k*_cat_, s^−1^	*k*_cat_/*K*_M_, μM^−1^ × s^−1^
KF	EAP	14 ± 4	(2.7 ± 0.2) × 10^−2^	(1.9 ± 0.5) × 10^−3^
	AP ^1^	19 ± 5	(2.1 ± 0.1) × 10^−3^	(1.1 ± 0.3) × 10^−4^
	T ^1^	0.77 ± 0.32	(2.7 ± 0.2) × 10^−2^	(3.5 ± 1.5) × 10^−2^
RBpol ^2^	EAP	–	–	(4.0 ± 0.3) × 10^−6^
	AP ^1^	110 ± 30	(2.7 ± 0.3) × 10^−2^	(2.5 ± 0.7) × 10^−4^
	T ^1^	0.35 ± 0.09	(2.7 ± 0.1) × 10^−2^	(7.7 ± 1.9) × 10^−2^
POLβ	EAP (primer-template)	190 ± 30	(6.7 ± 0.4) × 10^−3^	(3.5 ± 0.6) × 10^−5^
	EAP (gap)	81 ± 14	(4.2 ± 0.2) × 10^−3^	(5.2 ± 0.9) × 10^−5^
	THF or AP (primer-template)	negligible ^3^		
	AP (gap) ^1^	2.9 ± 1.5	(4.4 ± 0.2) × 10^−4^	(1.5 ± 0.8) × 10^−4^
	T (primer-template) ^1^	4.4 ± 1.7	(9.7 ± 0.7) × 10^−2^	(2.2 ± 0.9) × 10^−2^
	T (gap) ^4^	0.9 ± 0.2	0.69 ± 0.01	0.77 ± 0.17

^1^ From [34]; substrates with the same nucleotide context as in this study. ^2^ Enzyme saturation by the EAP substrate could not be achieved; *k*_cat_/*K*_M_ determined from the linear slope of the *v*_0_ vs. [S] dependence. ^3^ Incorporation of dAMP opposite AP or THF by POLβ is very low in the primer-template system without a downstream strand [34,36]. ^4^ From [39].

**Table 2:** Kinetic parameters of cleavage of THF- and EAP-containing substrates by AP endonucleases.

Enzyme	*K*_M_, nM		*k*_cat_, min^−1^		*k*_cat_/*K*_M_, nM^−1^ × min^−1^	
	THF:A	EAP:A	THF:A	EAP:A	THF:A	EAP:A
APEX1	11 ± 2	15 ± 6	25 ± 1	43 ± 5	2.3 ± 0.4	2.9 ± 1.2
Nfo	38 ± 8	11 ± 2	1.8 ± 0.1	2.6 ± 0.2	0.05 ± 0.01	0.24 ± 0.05
Xth	21 ± 8	12 ± 4	73 ± 7	180 ± 20	3 ± 1	15 ± 5

**Table 3:** Oligonucleotides used in this study.

Oligonucleotide	Sequence, 5′→3′	Modification
modified	CTCTCCCTTCXCTCCTTTCCTCT	X = EAP, THF or T
complementary	AGAGGAAAGGAGNGAAGGGAGAG	N = A, C, G or T
primer	[Fluo]AGAGGAAAGGAG	fluorescein
downstream	GAAGGGAGAG	

## Data Availability

All data are contained in the paper and Supplementary Materials.

## Supplementary Materials

The supporting information can be downloaded at: https://www.mdpi.com/article/10.3390/ijms232113353/s1.
