# Supplementary material for: Recognition of a Clickable Abasic Site Analog by DNA Polymerases and DNA Repair Enzymes
Source: Int J Mol Sci. 2022 Nov 1;23(21):13353. doi: 10.3390/ijms232113353 (PMC9655677; doi:10.3390/ijms232113353)
Supplement: Supplementary file 1 [file ijms-23-13353-s001.zip › ijms-1987004-supplementary.pdf]

SUPPLEMENTARY MATERIALS FOR

Recognition of a clickable abasic site analog by DNA polymerases and DNA repair enzymes

Anton V. Endutkin [1], Anna V. Yudkina [1], Timofey D. Zharkov [1], Daria V. Kim [1,2] and Dmitry O. Zharkov [1,2,*]

[1]SB RAS Institute of Chemical Biology and Fundamental Medicine, 8 Lavrentieva Ave., Novosibirsk 630090, Russia

[2]Department of Natural Sciences, Novosibirsk State University, 2 Pirogova Street, Novosibirsk 630090, Russia

[*]Corresponding authors: Anton V. Endutkin and Dmitry O. Zharkov

E-mail: aend@niboch.nsc.ru, dzharkov@niboch.nsc.ru

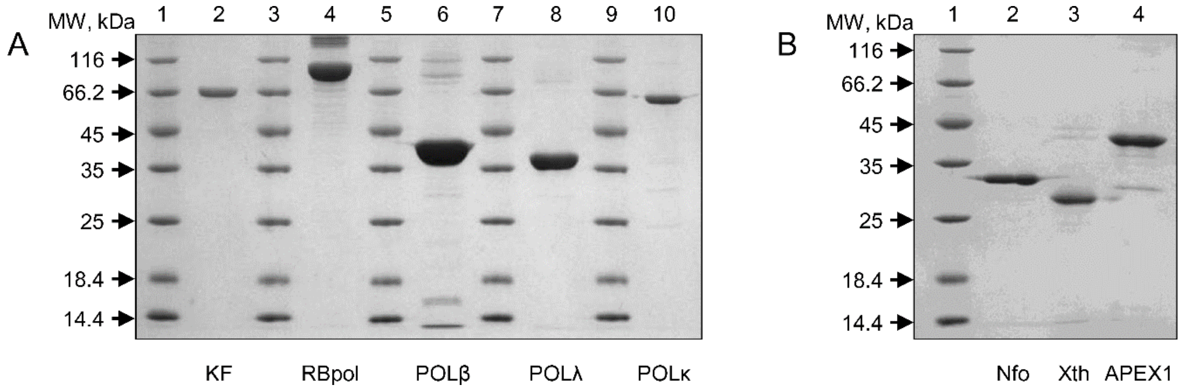

Supplementary Figure S1. Coomassie Blue-stained gels after 12% SDS-PAGE of the enzyme preparations used in this study. *Panel A*, DNA polymerases. Lanes: *1*, *3*, *5*, *7*, *9*: molecular weight markers (Pierce™ Unstained Protein MW Marker, Thermo Fisher Scientific, Waltham, MA, USA); *2*, KF; *4*, RBpol; *6*, POLβ; *8*, POLλ; *10*, POLκ. *Panel B*, AP endonucleases. Lanes: *1*, molecular weight markers; *2*, Nfo; *3*, Xth; *4*, APEX1.

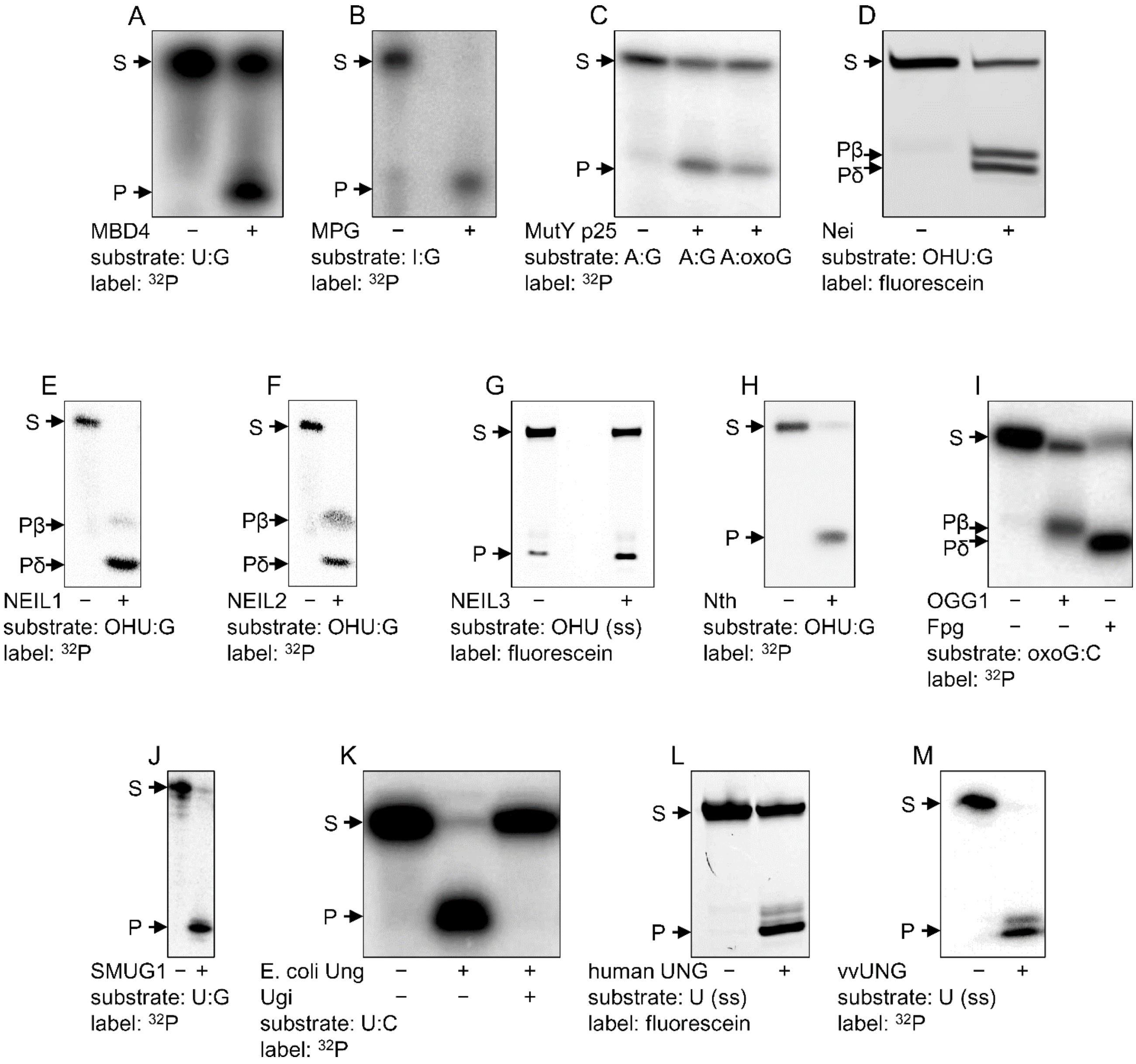

Supplementary Figure S2. Activity of the studied DNA glycosylases on their preferred base-containing substrates. The enzymes and the substrates are indicated in the panels. Arrows indicate: S, substrate; P, product. In *panels D*, *E*, *F*, and *I*, arrows Pβ and Pδ indicate products of β-elimination and δ-elimination, respectively. In *panel K*, the reaction was also carried out in the presence of 0.1 U/μl uracil–DNA gycosylase inhibitor (Ugi) from *Bacillus subtilis* bacteriophage PBS1 (New England Biolabs, Ipswich, MA, USA).
